# Laparoscopic redo surgery for sigmoid volvulus following laparoscopic sigmoidectomy

**DOI:** 10.1186/s40792-024-01961-3

**Published:** 2024-06-28

**Authors:** Hideyuki Masui, Kenji Kawada, Susumu Inamoto, Toshiaki Wada, Yoshiharu Sakai, Kazutaka Obama

**Affiliations:** 1 Departments of Surgery, Graduate School of Medicine, Kyoto University, 54 Shogoin- Kawahara-Cho, Sakyo-Ku, Kyoto, 606-8507 Japan; 2 Departments of Surgery, Hirakata Kohsai Hospital, Osaka, Japan; 3 Department of Surgery, Kurashiki Central Hospital, 1-1-1, Miwa, Kurashiki, Okayama, 710-8602 Japan; 4 Department of Surgery, Japanese Red Cross Osaka Hospital, Osaka, Japan; 5 Department of Surgery, Faculty of Medicine, Kindai University, Osaka, Japan

**Keywords:** Minimally invasive surgery, Laparoscopic redo surgery, Sigmoid volvulus, Sigmoidectomy

## Abstract

**Background:**

Sigmoid volvulus (SV) is an acute abdominal condition characterized by torsion of the sigmoid colon around the mesentery, and often results in intestinal obstruction that may progress to bowel ischemia, necrosis, or perforation. Although SV commonly occurs due to predisposing factors like anatomic variations, age-related motility disorders, chronic constipation, and neurologic diseases, its incidence following sigmoid colon cancer surgery has rarely been reported. Herein, we report a rare case of recurrent SV following laparoscopic sigmoidectomy, which was successfully treated by laparoscopic redo surgery.

**Case presentation:**

The patient was a 77-year-old man who had previously undergone laparoscopic sigmoidectomy for sigmoid colon cancer. Sixteen months postoperatively, he developed an incisional hernia at the umbilical site, which was treated with a laparoscopic repair using an intraperitoneal onlay mesh. After the hernia surgery, the patient had no anastomotic leakage or stenosis on regular follow-ups. However, 65 months after the first surgery, he presented with abdominal pain and distension. A computed tomography revealed that the remnant sigmoid colon was distended in a twisting manner around the anastomosis, leading to the diagnosis of SV. Although endoscopic de-torsion was successful, the SV recurred 2 months later, requiring elective laparoscopic redo surgery. The procedure involved resection of the sigmoid colon including the prior anastomosis with a left pararectal incision and DST re-anastomosis using a 25-mm circular stapler. The operation lasted 165 min with minimal bleeding and no complications. The postoperative course was uneventful. Pathological analysis confirmed fibrosis without malignancy. The patient remains well without recurrence of SV and anastomotic stenosis more than 5 years after surgery.

**Conclusion:**

SV following sigmoid colon cancer surgery has rarely been reported. This case illustrates the potential need for prophylaxis against postoperative SV, especially in patients with long sigmoid colon undergoing laparoscopic surgery for colorectal cancer. Further, laparoscopic redo surgery following initial laparoscopic surgery for colorectal cancer can be performed with minimal invasiveness, especially if patient selection is properly managed.

**Supplementary Information:**

The online version contains supplementary material available at 10.1186/s40792-024-01961-3.

## Background

Sigmoid volvulus (SV) is an acute abdomen characterized by torsion of the sigmoid colon around the mesentery. This condition often results in intestinal obstruction that may progress to bowel ischemia, necrosis, or perforation, requiring immediate medical attention. SV frequently occurs due to certain predisposing factors, such as anatomic variations, age-related motility disorders, chronic constipation, and neurologic diseases [1]; however, the incidence of SV following sigmoid colon resection is rare.

In this report, we present a noteworthy case of a patient who developed recurrent SV following laparoscopic sigmoidectomy for sigmoid colon cancer. This case was successfully treated by laparoscopic redo surgery, underscoring the prevention strategies for postoperative SV and the evolving role of minimally invasive surgery (MIS) in managing complex postoperative complications.

## Case presentation

**Fig. 1:**
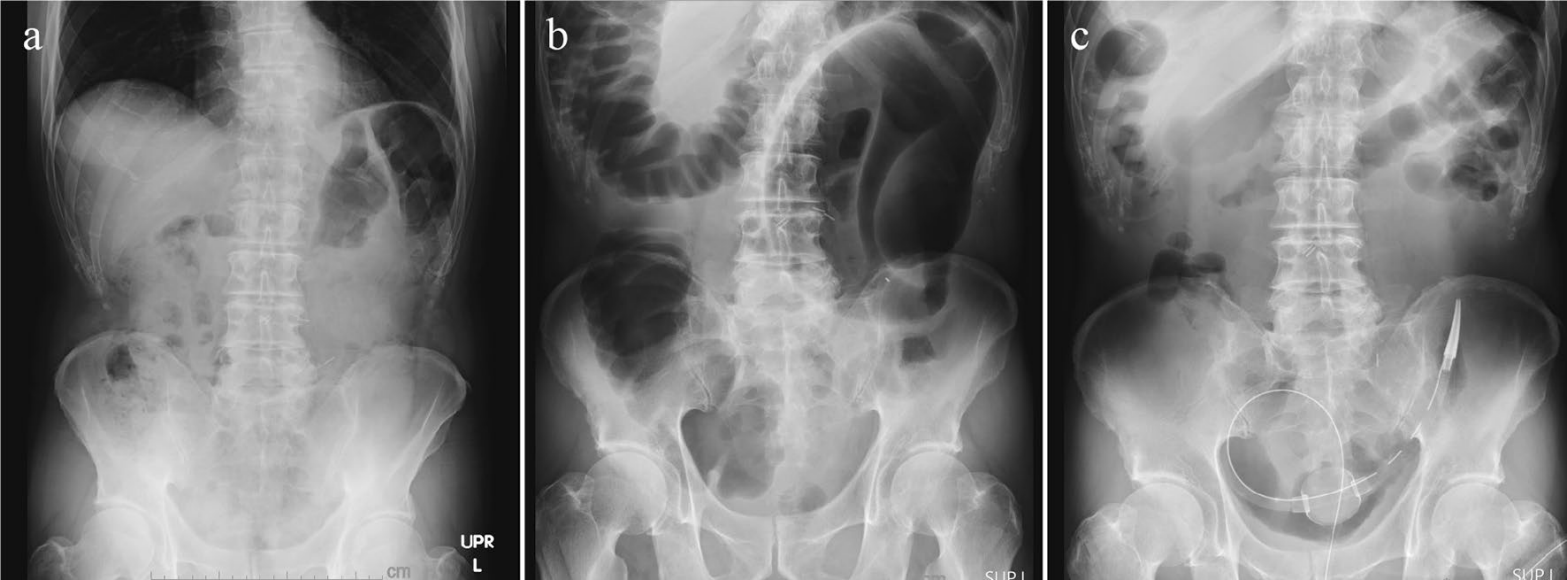
The sequence of X-rays (**a**–**c**) represents the following conditions: **a** baseline with no particular symptoms, **b** onset of sigmoid volvulus occurring 65 months after the initial surgery, and **c** pre-redo surgery after placement of a trans-anal decompression tube

**Fig. 2:**
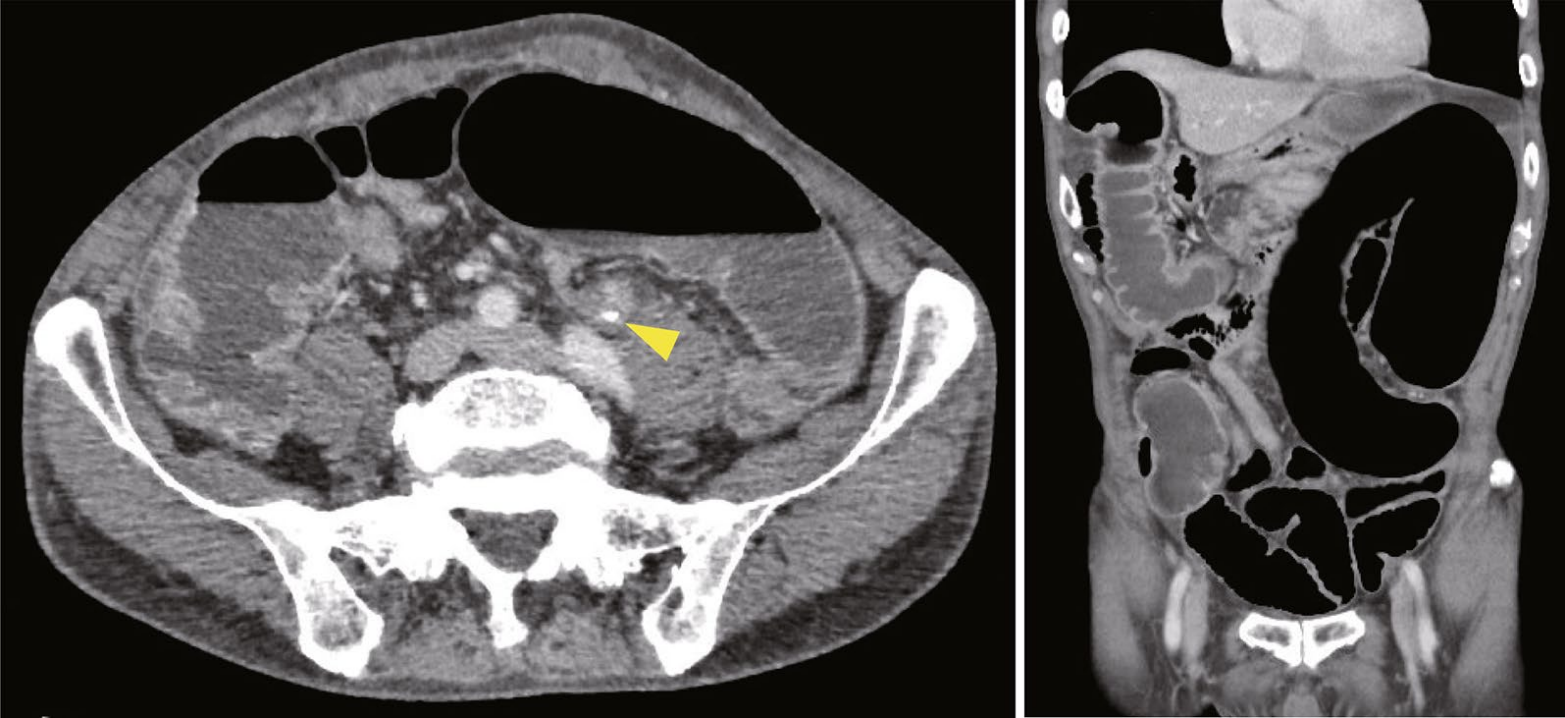
CT images from the time of the onset of sigmoid volvulus demonstrated the sigmoid colon twisted and distended around the anastomotic site. On the left is the axial view and on the right is the coronal view. Yellow arrowheads indicate the previous DST anastomotic site

**Fig. 3:**
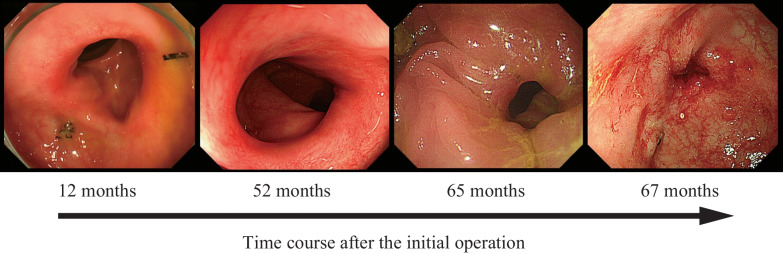
Colonoscopy showed the progressive stenosis due to anastomotic stricture after the initial operation (12, 52, 65, and 67 months later). Note that the anastomotic stenosis became more severe following an episode of sigmoid volvulus that occurred 65 months after the initial surgery

The patient was a 77-year-old man who had undergone laparoscopic sigmoidectomy with D3 lymph node dissection for locally advanced sigmoid colon cancer 69 months earlier. The inferior mesenteric artery was ligated at the root. For anastomosis, a double stapling technique (DST) was performed using a 60-mm linear stapler and a 25-mm circular stapler (Supplementary material 1). The length of resected sigmoid colon was approximately 21 cm. The patient recovered uneventfully with no leakage from the anastomosis. The final pathological stage was pT4aN0M0. Sixteen months postoperatively, the patient developed a hernia at the umbilical incision, which was treated with laparoscopic repair using an intraperitoneal onlay mesh. There were no complaints of symptoms at the outpatient clinic, and both radiographic and endoscopic findings were normal (Figs. 1a and 3). Postoperative endoscopy had been performed as a regular follow-up, and 52 months after the first operation, the anastomosis was still intact with no stenosis (Fig. 3). However, 65 months after the first operation, the patient presented with abdominal pain and distension, and radiographic examination revealed a distended large bowel (Fig. 1b). Computed tomography (CT) showed that the remnant sigmoid colon was twisted and distended around the previously stapled anastomosis (Fig. 2), leading to the diagnosis of SV. Because the colonoscope passed through the anastomosis without any problem, only de-torsion was performed without dilation (Fig. 3) and then the patient was discharged without complications. Nevertheless, he returned 2 months later with recurring abdominal pain and was diagnosed as a recurrent SV. This recurrence was again managed endoscopically. Endoscopic examination indicated progressive anastomotic stenosis with no signs of recurrence (Fig. 3). Consequently, he opted for elective reoperation to alleviate recurrent symptoms.

**Fig. 4:**
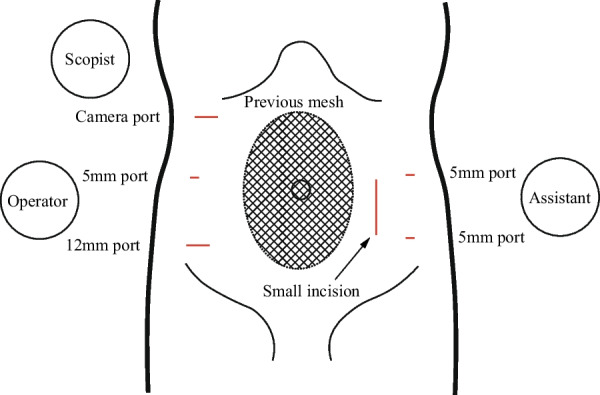
Port placement was arranged due to the presence of intraperitoneal mesh placed at the umbilical incision. A small incision was made in the left rectus abdominis muscle

**Fig. 5:**
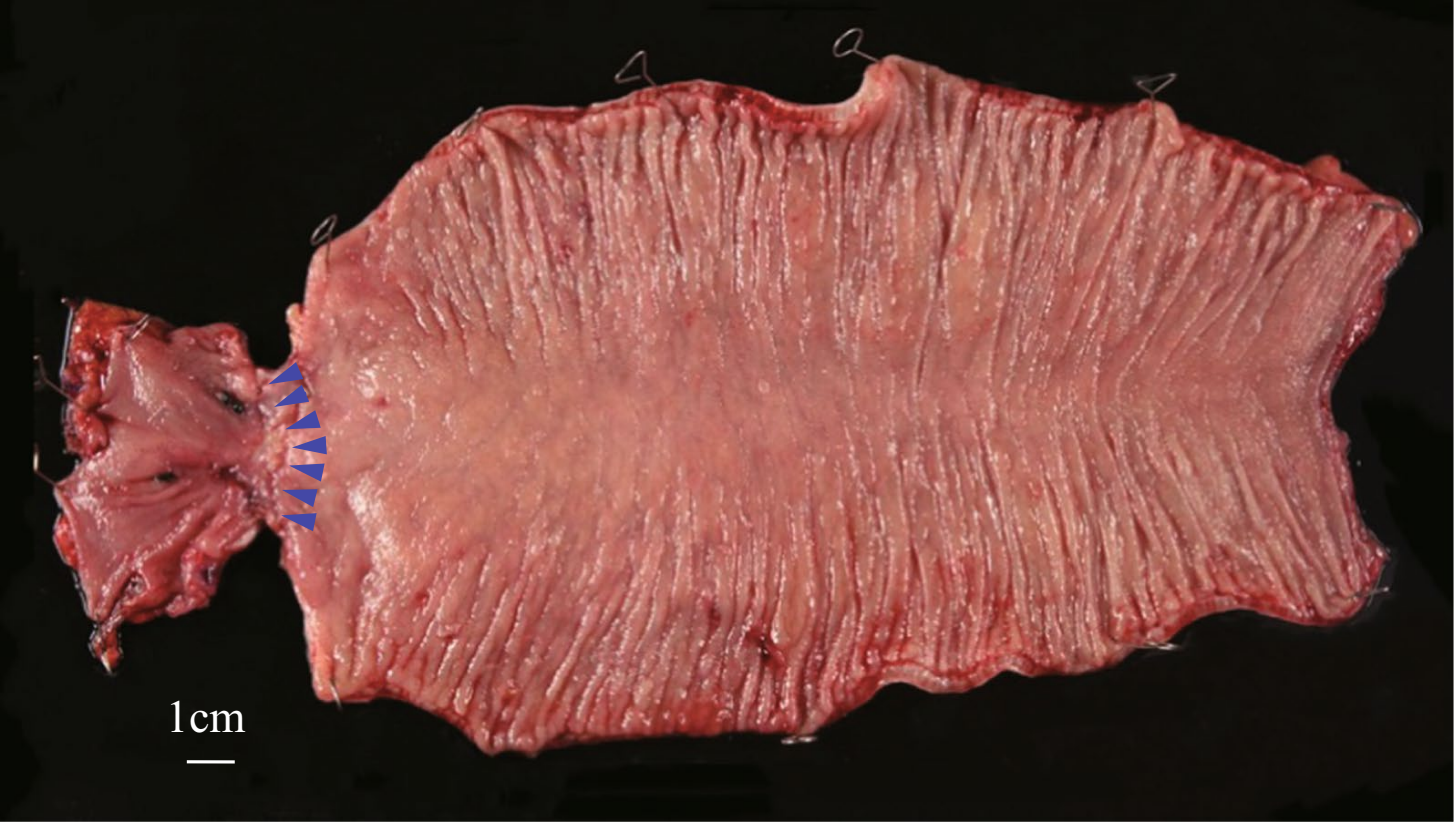
Specimen removed by laparoscopic redo sigmoidectomy. The blue dot line indicates the prior DST anastomosis. Pathological examination revealed fibrosis at the anastomotic site with no malignancy

Preoperatively, a decompression tube was placed transanally (Fig. 1c). Laparoscopic resection and re-anastomosis of the sigmoid colon, including the previous anastomosis, were then performed. Owing to the postoperative condition following the abdominal wall hernia operation, the camera port was positioned in the right subcostal area, avoiding the umbilical region (Fig. 4). Intraoperative observation revealed slight postoperative adhesions, but dissection of the adhesions between the omentum and the abdominal wall was feasible (Supplementary material 2). Furthermore, re-dissection of a previously dissected retroperitoneal area could be successfully performed with careful dissection and maintenance of the correct membrane structure. After complete mobilization of the remaining sigmoid colon and further dissection of the posterior rectal space, the rectum was transected on the distal side of the previous anastomosis using a stapler. A left pararectal incision was made and reconstruction was performed with DST anastomosis with a 25-mm circular stapler. The distance from the anal verge to the anastomosis site in the redo surgery was 10 cm. The operation time was 165 min, with minimal bleeding and no complications. Pathological analysis of the resected specimen revealed fibrosis at the anastomotic site without malignancy (Fig. 5). The length of resected colon was approximately 30 cm. The postoperative course was uneventful. Five years post-surgery, the patient remains well with no recurrence of SV and anastomotic stenosis.

## Discussion

SV is one of the common causes of colonic obstruction, accounting for 3–5% of intestinal obstruction, especially in older patients [2]. Complicated cases typically require urgent colonoscopy for diagnosis and treatment [3, 4]. If initial endoscopic decompression is ineffective, a surgical approach should be considered. In the past, laparotomy with resection of the redundant sigmoid colon was considered the gold standard treatment. However, with the widespread adoption of laparoscopic surgery, this approach is increasingly being applied to SV [5].

SV occurring following sigmoidectomy is rare, mainly due to the shortening of the sigmoid colon as a result of sigmoid colon resection. In the literature, there has been only one other reported case of a patient developing complicated SV with necrosis following sigmoidectomy for sigmoid colon cancer, which required emergent laparotomy with colostomy [6]. However, bloating is one of common complaints after sigmoidectomy for cancer [7], and postoperative SV might be under-recognized.

In our case, the patient's long colonic length and the high anastomosis may have contributed to disease development, as the initial surgery (Supplementary material 1) shows the remnant colon was redundant. It was also reported that DST anastomosis in laparoscopic surgery tends to preserve the oral intestine longer than necessary compared to open laparotomy [8]. Endoscopic findings (Fig. 3) suggest the first SV caused ischemia of the anastomosis, which in turn caused the rapid progression of anastomotic stenosis. In cases of long sigmoid colon, preventive measures against postoperative SV may include the following points: (1) resection of a longer bowel than usual; and (2) fixation of the remnant colon to the abdominal wall with a few stitches. Additionally, although not applied in the presented case, intraoperative ICG fluorescence angiography would be also a valuable tool for identifying insufficient blood flow during surgery [9].

Anastomotic stricture after stapled colorectal anastomosis is a complication seen in approximately 2% in a multicenter retrospective study; however, the stricture usually responds to endoscopic dilation and rarely requires reoperation [10]. In our case, preoperative transanal decompression was performed to ensure a good surgical field for a laparoscopic approach. The effectiveness of this strategy was validated in a recent report [11].

To our knowledge, this is the first case treated by laparoscopic redo surgery for SV following laparoscopic resection of sigmoid colon cancer. The intraoperative findings in this case showed minimal intra-abdominal adhesions owing to the two previous laparoscopic operations. Re-anastomosis was successfully performed, avoiding a colostomy that would have affected the patient’s quality of life. Laparoscopic redo surgery following initial laparoscopic surgery for colorectal cancer remains a topic of debate. Given the risk of redo surgery involving resection and re-anastomosis, laparotomy is traditionally preferred for complicated reoperations [12]. However, advances in laparoscopic surgery have made this technique applicable to complex cases. Recent studies have demonstrated that repeated laparoscopic colorectal resection is feasible and safe when performed with a skilled technique, although dense postoperative adhesions and anatomical structural changes can present challenges in MIS [13]. Therefore, careful preoperative assessment and meticulous surgical techniques are crucial for safe laparoscopic redo surgery. If possible, this would provide the benefits of MIS: reduced blood loss, less wound pain, quicker recovery, and decreased incidence of ileus and surgical-site infections.

## Conclusion

We report a rare case of recurrent SV following sigmoidectomy. Laparoscopic redo surgery following initial laparoscopic surgery for colorectal cancer can provide minimal invasiveness, especially if patient selection is properly managed. In cases with long sigmoid colon, it may be necessary to consider prophylaxis for postoperative SV.

### Supplementary Information

Supplementary material 1. Operative video of the first operation (laparoscopic sigmoidectomy for sigmoid colon cancer). A video related to anastomosis is presented.

Supplementary material 2. Operative video of laparoscopic redo surgery. After the sigmoid mesentery was pulled toward the anus, the previous anastomotic site could be seen with no obvious adhesions around it. We then proceeded to re-dissect the previously dissected retroperitoneal area. After progressing with the internal approach, we switched to an external approach. Dissection was carried out up to the area near the splenic flexure. Then, we proceeded with additional dissection in the posterior rectal space. The rectum was transected with a surgical stapler on the distal side of the previous anastomosis. The reconstruction was performed using a double-staple technique, ensuring a secure and tension-free anastomosis.

## Data Availability

Data sharing is not applicable to this article as no datasets were generated or analyzed during the current study.

## Abbreviations

SV: Sigmoid volvulus
MIS: Minimally invasive surgery
DST: Double stapling technique
CT: Computed tomography
